# Primary subcutaneous hydatid cyst in the left flank: A case report and review of the literature

**DOI:** 10.1016/j.ijscr.2025.111206

**Published:** 2025-03-25

**Authors:** Atabak Sedigh-Namin, Sajad Ghadimpour, Elmira Mousavi, Sina Seifimansour, Alireza Bagheri Toularoud

**Affiliations:** a Students Research Committee, School of Medicine, Ardabil University of Medical Sciences, Ardabil, Iran; b Department of Surgery, School of Medicine, Ardabil University of Medical Sciences, Ardabil, Iran; c Department of Anatomical Sciences and Pathology, School of Medicine, Dr. Fatemi Hospital, Ardabil University of Medical Sciences, Ardabil, Iran; d Cancer Immunology and Immunotherapy Research Center, Ardabil University of Medical Sciences, Ardabil, Iran

**Keywords:** Hydatid cysts, Subcutaneous, Case report

## Abstract

**Introduction and importance:**

Hydatidosis, a major parasitic disease in Mediterranean countries, primarily affects the liver and lungs, but in rare cases, it may also affect the subcutaneous tissue. Subtle symptoms often delay diagnosis. This report presents a rare case of left flank hydatid cyst.

**Case presentation:**

We report a 40-year-old man who had been suffering from a painless mass in the left flank for three months, which was recently painful, and was diagnosed with a subcutaneous hydatid cyst. Imaging and serology tests were inconclusive. The intact cyst was surgically removed, and histology confirmed hydatid cysts. Postoperative albendazole therapy was given for three months and resulted in complete recovery.

**Clinical discussion:**

Hydatid cysts caused by Echinococcus granulosus primarily affect the liver and lungs, with subcutaneous cases being rare. This is the first reported case of a primary subcutaneous hydatid cyst in the left flank.

**Conclusion:**

Hydatid disease should be considered in the differential diagnosis of soft tissue tumors, particularly in endemic areas. Outside of typical liver or lung involvement, diagnosis can be challenging and often delayed.

## Highlights

• First reported case of a primary subcutaneous hydatid cyst in the flank region without involvement of other organs
• Importance of considering hydatid cysts in the differential diagnosis of soft tissue masses, especially in endemic regions
• Limitations of serological tests for intact cysts and the need for strong clinical suspicion in high-risk areas
• Multidisciplinary collaboration (radiology, pathology, surgery) is essential for effective management.

## 1 Introduction

This case report follows the SCARE criteria exclusively to ensure a structured and comprehensive presentation of the clinical scenario [1]. Hydatidosis is a parasitic infection and zoonotic disease primarily caused by *Echinococcus granulosus* [2]. Hydatid disease is common in various regions worldwide [2], including the Middle East. In Iran, hydatid cyst infection rates vary significantly, ranging from 0.22 % to 63.2 % in humans and up to 34.6 % in animals like camels. While higher rates are observed in urban areas, there is a lack of data for other Middle Eastern countries. [3]. It is endemic in Iran, with most cases reported from the northwestern region. *Echinococcus granulosus* primarily infects dogs and herbivores, serving as its definitive and intermediate hosts, respectively. Humans, considered incidental hosts, acquire the infection by consuming contaminated water or vegetables [4]. The parasite's eggs are spread through the feces of infected animals, facilitating transmission to other species [5]. The liver is the most frequently affected organ, followed by the lungs as the second most common site of infection. In rare cases, the disease can also involve other parts of the body [6]. Primary subcutaneous hydatid disease indicates the absence of a primary focus of hydatid infection. Solitary primary subcutaneous hydatid cysts are extremely rare, with an unknown incidence [8]. This rare disease can pose diagnostic and management challenges, especially for the inexperienced. Even in endemic regions, the diagnosis of a subcutaneous hydatid cyst often raises concerns about potential anaphylaxis or local/systemic recurrence. These lesions may remain asymptomatic for prolonged periods. Clinical manifestations are often nonspecific and related to the size, location, or complications of the cyst [9]. In our presentation, we describe the case of a 40-year-old male with a growing mass in the left flank.

## 2 Case presentation

**Fig. 1:**
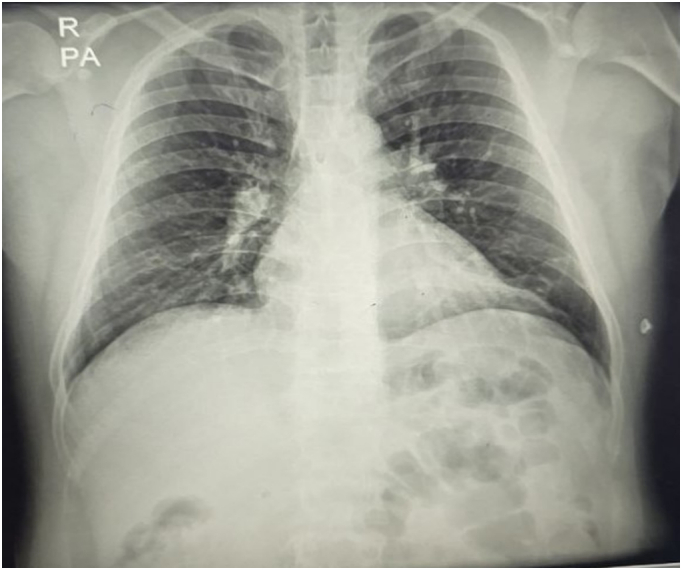
The chest X-ray image shows no abnormalities in the lungs or other organs.

**Fig. 2:**
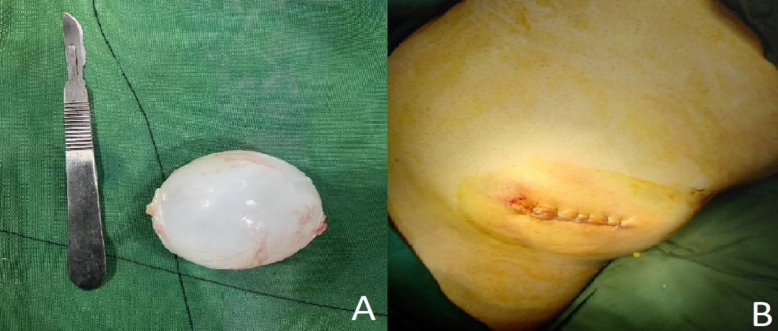
A. Image of the totally excised hydatid cyst. B. Postoperative view of the surgical site after closure (left flank of the patient).

**Fig. 3:**
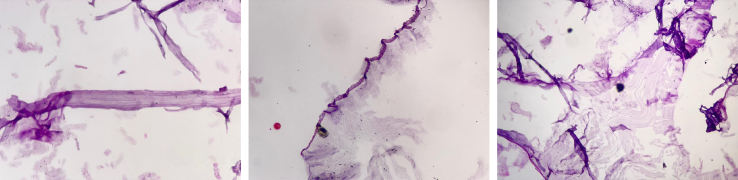
Cyst wall has 3 structural components: Outer acellular laminated membrane (1 mm thick) germinal membrane (a transparent nucleated lining) protoscolices, attached to the membrane and budding from it.

A 40-year-old male presented at our hospital with a three-month history of a non-painful mass in the left flank. Initially, the mass was asymptomatic, but with time, the pain increased. The patient's chief complaint on admission was an increase in pain in the mass. The physical examination confirmed the existence of a palpable subcutaneous mass. The skin over the mass appeared completely normal, with no signs of erythema, ulceration, or any previous trauma. The patient had no relevant dermatological condition. The patient did not have fever or chills, and the mass was not warm. A differential diagnosis of lipoma was clinically considered. An abdominal and pelvic ultrasound revealed a cystic tumor located subcutaneously on the left flank. It measured 7 cm in diameter at its largest position and had a volume of approximately 70 cc. No additional abdominal cystic lesions were found. The differential diagnosis included simple cysts and hydatid cysts. Because of how common hydatid disease is in the area, the mass's features and location made a hydatid cyst a likely diagnosis, even though there isn't much written about this happening. The chest X-ray (Fig. 1) and routine laboratory test (complete blood count) were normal as shown in Table 1. Computed tomography (CT) of the abdomen showed no significant abnormalities, and no cystic lesions were detected. Nevertheless, the cyst was located in the superficial layers of the abdominal wall, which was not clearly shown on the typical abdominal CT scan that mainly highlights internal organs. Although a chest CT scan is generally recommended to rule out pulmonary involvement in cases of suspected hydatid disease, the chest X-ray and CT scan performed in this case showed no abnormalities. No evidence of lung involvement or hydatid cysts in the pulmonary tissue was observed. The serological enzyme-linked immunosorbent test (ELISA) for Echinococcal antigens produced a negative result. The patient did not show any signs of infection. The immune system was healthy, and there were no indications of bacterial or fungal infections. Given the patient's clinical presentation and the strong suspicion of a hydatid cyst or lipoma, and considering the absence of neurological symptoms, brain imaging was not performed. Subcutaneous cysts in this region are typically not associated with neurological involvement unless there are specific clinical signs, which were not present in this case. The patient, exhibiting the specified characteristics and a strong clinical suspicion of a hydatid cyst, received a referral for an operation. The cyst had characteristics typical of a hydatid cyst, including a solid, multilocular wall. We meticulously resected the cyst from the surrounding tissue after accessing it to prevent its rupture. The site of the cyst was properly cleaned with hypertonic saline (3 % NaCl) to avoid any possible infection (Fig. 2). A sample of the cyst wall was dispatched to the pathology laboratory for additional analysis. A Histological examination after surgery revealed a hydatid cyst (Fig. 3). The cyst wall contained acellular lamellar deposits, surrounded by a thin germinal layer. Interestingly, protoscolices were visible in the histological images, which was consistent with the typical presentation of a hydatid cyst. This observation aligns with the clinical suspicion and the characteristics of the mass.

**Table 1:** Initial laboratory findings.

Laboratory study	Value	Reference range
WBC count	6.10 × 10^9/L	4–11 × 10^9/L
Neutrophils	53 %	40–75 %
Lymphocytes	33 %	20–45 %
Monocytes	7 %	2–9 %
Eosinophils	5 %	1–5 %
Basophils	2 %	0–2 %
RBC	4.50 × 10^12/L	4.1–5.1 × 10^12/L
Hemoglobin	15.7 g/dL	13.5–17.5 g/dL
Platelets	247 × 10^9/L	150–450 × 10^9/L
PT	12 s	
PTT	35 s	28–45 s
INR	1 s	1–1.3 s

g/dL: grams per deciliter; WBC: white blood cell; RBC: red blood cell; PT: prothrombin time; PTT: partial thromboplastin time; INR: international normalized ratio.

We monitored the patient clinically after surgery to ensure there were no problems related to the procedure or the medical treatment. We maintained the albendazole therapy protocol for three months. We conducted routine outpatient follow-up appointments to assess potential issues, including hepatic damage or gastrointestinal disorders, that could arise from the treatment. No abnormalities in liver function or other organs were detected.

## 3 Discussion

Hydatid cyst is a common disease in sheep-raising countries of the Middle East, Australia, Baltic states, South America, and the Mediterranean [[9], [10]]. Primary subcutaneous involvement is a rare occurrence in medical literature, with an incidence ranging from 0.2 % to 2 % [11]. The incidence of subcutaneous involvement in various anatomical regions, as reported in our literature (Table 2) [[12], [13], [14], [15], [16], [17], [18], [19], [20], [21], [22], [23], [24], [25], [26], [27], [28], [29], [30], [31], [32], [33], [34], [35], [36], [37], [38], [39], [40], [41], [42], [43], [44], [45], [46], [47], [48], [49], [50], [51], [52], [53], [54], [55]], is as follows: Thigh: 29.27 %, Gluteal: 9.76 %, Shoulder: 7.32 %, Abdominal wall: 4.88 %, and Lumbar: 4.88 %.

**Table 2:** Initial laboratory findings.

1st author (reference)	Year	Country	Patient age/sex	Cyst location	Symptoms	Treatment
Ben Khalifa [14]	2022	Tunisia	55/F	Para-vertebral lumbar	Painless mass	Surgery, post-operative albendazole for 3 months
Meltem Özdemir [15]	2020	Turkey	77/M	Gluteal	Painless mass	Pre-operative albendazole for 5 days, surgery, post-operative albendazole for 6 months
Al-Hakkak [16]	2018	Iraq	48/F	Hypochondrium	Nausea and vomiting	Surgery, post-operative albendazole for 3 months
Hekmatnia [17]	2018	Iran	59/F	Thigh	Painful mass	Surgery
Pascua L [18]	2017	Spain	77/F	Gluteal	Mobile and firm mass	Surgery, post-operative albendazole for 3 months
Chevalier [19]	1994	France	40/M	Thigh	Painful mass	Surgery, post-operative albendazole for 3 months
Voucharas [20]	1997	Greece	50/F	Thigh	Mobile and painless mass	Surgery
Ok [21]	2000	Turkey	12/F	Submandibular	Mobile and painful mass	Surgery, post-operative albendazole for 3 months
Ozturk [22]	2001	Turkey	20/M	Malar	Mobile and painless mass	Surgery
Acar [23]	2001	Turkey	42/F	Thigh	Mobile and painful mass	Surgery, post-operative albendazole for 3 months
Baldi [24]	2002	Italy	54/F	Scapula	Painless mass	Surgery
Arinc [25]	2003	Turkey	39/M	Sternum	Painless mass	Surgery, post-operative albendazole for 3 months
Orhan [26]	2003	Turkey	43/F	Thigh	Painful mass	Surgery, post-operative albendazole for 3 months
Guiral [27]	2004	Spain	34/F	Knee	Mobile and painless mass	Surgery, post-operative albendazole for 3 months
Koybasioglu [28]	2004	Turkey	22/F	Infraumblical	Painless mass	Surgery
Kiyak [29]	2006	Turkey	62/F	Inguinal	Painless mass	Surgery
Gurbuz [30]	2006	Turkey	10/F	Retroauricular	Fixed and painless mass	Surgery, post-operative albendazole for 3 months
Bedioui [31]	2007	France	70/F	Hypogastric	Painless mass	Surgery
Daoudi [32]	2007	France	21/F	Gluteal	Painless mass	Surgery
Demirel [33]	2007	Turkey	73/F	Subclavicular	Painless mass	Surgery
Dogmus [34]	2007	Turkey	21/F	Lumbar	Mobile and painless mass	Surgery, post-operative albendazole for 3 months
Safioleas [35]	2008	Greece	73/M	Gluteal	Mobile and painless mass	Surgery, post-operative albendazole for 3 months
Parsak [36]	2008	Turkey	29/F	Thigh	Fixed and painful mass	Surgery, post-operative albendazole for 3 months
Dirican [37]	2008	Turkey	64/M	Thigh	Mobile and painless mass	Surgery, post-operative albendazole for 3 months
Dirican [37]	2008	Turkey	67/M	Palm	Fixed and painless mass	Surgery, post-operative albendazole for 3 months
Gupta [38]	2008	India	12/F	Shoulder	Mobile and painless mass	Surgery, post-operative albendazole for 3 months
Gupta [38]	2008	India	20/M	Back	Mobile and painless mass	Surgery, post-operative albendazole for 3 months
Losanoff [39]	2004	USA	38/M	Axillary region	Painless, round, palpable mass	Surgery
Sãvulescu [40]	2010	Romania	46/F	Thigh	Painless, round, palpable mass	Surgery, post-operative albendazole for 3 months
Ozkan [41]	2010	Turkey	84/F	Thigh	Painless mass	Surgery, post-operative albendazole for 3 months
Singal [42]	2010	India	26/F	Thigh	Painless mass	Surgery, post-operative albendazole for 3 months
Battyany [43]	2011	Hungary	63/M	Popliteal fossa	Painless hypermic mass	Pre-operative albendazole, surgery, post-operative albendazole
Sallami [44]	2011	France	42/M	Lumbar	Painless mass	Surgery
Ousadden [45]	2011	Morocco	70/F	Abdominal wall	Mobile and painless mass	Surgery
Bansal [46]	2011	India	24/M	Face	Slowly growing mass	Surgery, post-operative albendazole for 6 weeks
Mushtaque [47]	2012	India	N/A	Gluteal	Palpable lump	Surgery, post-operative albendazole for21 days
Rais [48]	2012	France	58/F	Scalp	Palpable mass	Surgery
Abhishek [49]	2012	India	60/F	Abdominal wall	Painless mass	Surgery, post-operative albendazole for 3 months
Jarboui [50]	2012	Tunisia	53/F	Supraclavicular	Painful mass	Surgery, post-operative albendazole for 8 weeks
Ozdemir [51]	2012	Turkey	29/F	Shoulder	Painful mass	Surgery, post-operative albendazole for 4 weeks
Vecchio [52]	2013	Italy	68/M	Shoulder	Painless mass	Surgery, post-operative albendazole for 4 weeks
Burgazli [53]	2013	Germany	63/M	Abdominal wall	Palpable mass	Surgery, post-operative albendazole for 3 months
Almadani [54]	2013	Saudi Arabia	53/M	Thigh	Frim mass	Surgery
Yucesoy [55]	2013	Turkey	44/F	Thigh	Soft mass	Percutaneous treatment
Haslak [56]	2014	Turkey	37/F	Lumbar	Palpable mass	Surgery, post-operative albendazole for 3 months
Şakir Ekşi [57]	2014	Turkey	62/F	Thoracic	Palpable mass	Surgery, post-operative albendazole for 3 months
Present case	2024	Iran	40/M	Flank	Painful mass	Surgery, post-operative albendazole for 3 months

Abbreviations: M: male, F: female, N/A: not available.

The present instance is the first reported case of primary subcutaneous hydatid disease of the flank, occurring without involvement of other organs, indicating the cyst's primary origin.

The exact mechanism underlying primary subcutaneous localisation is unclear. Two plausible pathways have been proposed: (a) direct contamination of the subcutaneous tissue through compromised skin integrity, or (b) subcutaneous colonisation following the ingestion of eggs, which subsequently traverse the liver and lungs before reaching the subcutaneous tissue [56,57].

The hydatid cyst enlarges gradually, leading to clinical manifestations that vary depending on the organ affected and its tolerance. In cases of liver and lung involvement, symptoms typically emerge at a later stage, whereas brain or eye involvement often results in earlier detection [9]. Clinical presentation varies based on the cyst's size, location, and condition. It is usually presented as a non-inflammatory and painless mass. The mortality rate associated with hydatid disease is approximately 4 % [23].

In endemic regions, differential diagnosis for cystic lesions should include echinococcosis. Other conditions to consider in the differential diagnosis include aneurysms, tuberculosis, haematomas, benign cysts, mycoses, benign or malignant neoplasms, and abscesses [[9], [10]].

Diagnostic modalities employed include radiographic imaging and immunohistochemical techniques [18]. Ultrasound (US) is a valuable tool for determining the size, location, type, and diagnosis of cysts. Additionally, computed tomography (CT) plays an essential role in assessing adjacent organ involvement and aiding surgical planning. Also, imaging tests like magnetic resonance imaging (MRI), computed tomography (CT), ultrasound (US), and sometimes just plain radiography are very important for looking at cysts in different organs. Certain x-ray features, like daughter cysts, calcifications on the cyst wall, and detached germinal membranes, help doctors figure out what's wrong. Among these, an MRI offers superior visualisation, particularly for detailed cutaneous imaging [9,12,22]. In our case, we utilised US and chest X-rays. However, since the US images were inaccessible, we relied on the US report for documentation in this article.

ELISA and indirect hemagglutination tests are commonly used for serum screening in cystic echinococcosis. However, serological results can be unreliable in certain scenarios. Cysts that are whole and not broken don't release antigens, so they don't cause immune system responses. This can cause false-negative serological results [58,59].

In our case, the ELISA test result was also negative.

Complete surgical resection is the primary treatment for hydatid cysts, and it requires meticulous execution to prevent cyst rupture. Maintaining the integrity of the cyst wall during resection is crucial to prevent disease dissemination and anaphylaxis. If it's not possible to remove the cyst completely, the contents should be aspirated during surgery, and then the area should be irrigated with scolicidal agents. Finally, the empty cyst should be removed. Surgical methods like pericystectomy, endocystectomy, marsupialization, capitonage, simple drainage, or organ resection are often used, depending on the type of cyst and where it is located [9]. However, certain factors such as multiple cysts, pregnancy, unsuitable medical conditions, or patient refusal may contraindicate surgery. In such cases, less invasive methods like injection, puncture, aspiration, and reaspiration (PAIR) or medical therapy are alternatives [10].

Medical treatment with benzimidazole compounds, particularly albendazole (10–15 mg/kg/day), has proven effective [10]. Albendazole is preferred over mebendazole due to its superior pharmacokinetics. Treatment that lasts at least three months is linked to good results, such as a full recovery in one-third of patients and a significant decrease in cyst size in 30–50 % of cases. Surgery and albendazole therapy together have been shown to lower the chance of hydatid cystic disease coming back after surgery [[9], [10]]. In our case, we treated the patient with surgery and a 3-month course of albendazole.

## 4 Conclusion

Hydatid disease should be included in the differential diagnosis of soft tissue tumors, especially in endemic regions. Subcutaneous hydatid cysts are very rare and are likely spread through hematogenous routes, though the exact mechanism remains unclear. Diagnosis can be challenging, especially in cases involving organs other than the liver or lungs, often leading to delayed treatment. When a subcutaneous cystic lesion is identified, hydatid disease must be considered. The optimal treatment involves en bloc surgical excision followed by benzimidazole therapy to prevent recurrence. Future clinical implications include the need for increased awareness and better diagnostic tools for early detection of hydatid cysts, especially in endemic areas. Clinicians should consider the potential for hydatid disease when evaluating unusual soft tissue masses. Additionally, further research is needed to understand the pathophysiology of subcutaneous hydatid cysts and explore more effective treatment strategies.

## CRediT authorship contribution statement

Atabak Sedigh-namin: Data collection, data analysis, and manuscript writing, and first author.
Alireza Bagheri Toularoud: Study design, data interpretation, and manuscript review, and first author.
Sajad Ghadimpour: Study concept and design, data interpretation, and manuscript review, and corresponding author.
Elmira Mousavi: Data collection, data analysis.
Sina Seifimansour: Data collection, data analysis.

## Consent

Written informed consent was obtained from the patient for publication of this case report and accompanying images. A copy of the written consent is available for review by the Editor-in-Chief of this journal on request.

## Ethical approval

The study was reviewed and approved by the relevant Research Ethics Committee in accordance with ethical guidelines.

## Guarantor

Alireza Bagheri Toularoud accepts full responsibility for the work and the conduct of the study, had access to the data, and controlled the decision to publish.

## Research registration number

1. Name of the registry: None
2. Unique identifying number or registration ID: None
3. Hyperlink to your specific registration: None.

## Funding

None.

## Declaration of competing interest

Authors have no conflict of interest to declare.

## References

[1] Sohrabi C., Mathew G., Maria N., Kerwan A., Franchi T., Agha R.A. (2023). The SCARE 2023 guideline: updating consensus Surgical CAse REport (SCARE) guidelines. Int J Surg Lond Engl..

[2] Rawat S., Kumar R., Raja J., Singh R.S., Thingnam S.K.S. (2019). Pulmonary hydatid cyst: review of literature. J. Family Med. Prim. Care.

[3] Seyedsadeghi M., Ghobadi J., Haghshenas N., Habibzadeh A. (2019). Gluteal hydatid cyst: a case report. Iran. J. Parasitol..

[4] Woolsey I.D., Miller A.L. (2021). Echinococcus granulosus sensu lato and Echinococcus multilocularis: a review. Res. Vet. Sci..

[5] Garg M.K., Sharma M., Gulati A., Gorsi U., Aggarwal A.N., Agarwal R. (2016). Imaging in pulmonary hydatid cysts. World J. Radiol..

[6] Andalib Aliabady Z., Berenji F., Jamshidi M.R. (2015). A case report of muscle hydatidosis from Iran. Iran. J. Parasitol..

[8] Di Cataldo R., Latino A., Cocuzza G. (2009). Li Destri, Unexplainable development of a hydatid cyst. World J. Gastroenterol..

[9] Doty J.E., Tompkins R.K. (1989). Management of cystic disease of the liver. Surg. Clin. North Am..

[10] Moro P., Schantz P.M. (2009). Echinococcosis: a review. Int. J. Infect. Dis..

[11] Kayaalp, Blumgart L.H., Belghiti R.J., DeMatteo R.P., Chapman W.C., Büchler M.W., Hann L.E., D’ Angleca M. (2007). Surgery of Liver Biliary Tract and Pancreas.

[12] Ben Khalifa M., Ghannouchi M., Hammouda S., Taboubi W., Omri A., Nacef K., Boudokhane M. (Nov 1 2022). Primary subcutaneous hydatid cyst: an exceptional location. IDCases.

[13] Özdemir M., Kavak R.P., Kavak N., Akdur N.C. (Feb 11 2020). Primary gluteal subcutaneous hydatid cyst. IDCases.

[14] Al-Hakkak S.M.M. (2018). Isolated primary subcutaneous hydatid in right hypochondrium region: case report. Int. J. Surg. Case Rep..

[15] Hekmatnia F., Motififard M., Yazdi H.A., Rizi A.M., Hedayat P., Hekmatnia A. (Mar 27 2018). A large primary subcutaneous hydatid cyst in proximal thigh: an unusual localization. Adv. Biomed. Res..

[16] Ramos Pascua L, Santos Sanchez JA, Samper Wamba JD, Alvarez Castro A, Rodriguez Altonaga J. Atypical image findings in a primary subcutaneous hydatid cyst in the gluteal area. Radiography (Lond). 2017 Aug;23(3):e65-e67. doi: 10.1016/j.radi.2017.03.017. Epub 2017 Apr 26. PMID: 28687303. 28687303

[17] Chevalier X., Rhamouni A., Bretagne S., Martigny J., Larget-Piet B. (Sep 1994). Hydatid cyst of the subcutaneous tissue without other involvement: MR imaging features. AJR Am. J. Roentgenol..

[18] Voucharas C., Papaioannidis D., Papamichael K. (May 1998). Subcutaneous mass of the right thigh. Postgrad. Med. J..

[19] Ok E., Sözüer E.M. (May 2000). Solitary subcutaneous hydatid cyst: a case report. Am. J. Trop. Med. Hyg..

[20] Oztürk S., Devec M., Yildirim S. (Feb 2001). Hydatid cyst in the soft tissue of the face without any primary. Ann. Plast. Surg..

[21] Acar T., Tacyildiz R., Tuncal S. (2001). Isolated hydatid cyst in the subcutaneous tissue. Turk. J. Med. Sci..

[22] Baldi A., Rossiello L., Rossiello R., Baldi F. (2002). Echinococcal cysts with primary cutaneous localization. Br. J. Dermatol..

[23] Arinc S., Alper L., Arinc B. (2003). Hydatid disease of chest wall: a case report. Gogus Hastalik Derg.

[24] Orhan Z., Kara H., Tuzuner T., Sencan I., Alper M. (2007). Primary subcutaneous cyst hydatid disease in proximal tight. BMC Musculoskelet. Disord..

[25] Guiral J., Rodrigo A., Tello E. (2004). Subcutaneous echinococcosis of the knee. Lancet.

[26] Koybasioglu F., Arikok A., Ozturk E., Onal B.U. (2004). Fine needle aspiration cytology in diagnosis of a subcutaneous echinococcal cyst. Acta Parasitol..

[27] Kiyak G., Ozer M., Aktimur R., Kusdemir A. (2006). Primary hydatid disease of the soft tissue. Int. J. Surg..

[28] Gurbuz M.K., Ozudogru E., Calki H., Kabukcuoglu S., Dogan N. (2006). A retroauricular situated primary hydatid cyst: a case report. Turk. Arch. Otolaryngol..

[29] Bedioui H., Makni A., Nouira K., Mekni A., Daghfous A., Ayadi S. (2007). Subcutaneous hydatid cyst. Case report of an exceptional location. Med. Trop..

[30] Daoudi A., ElibrahimiA Loudiyi, Elmrini A., Chakour K., Boutayeb F. (2006). Solitary subcutaneous hydatid cyst of gluteal area: an unusual lacalization. An case report. Ann. Chir. Plast..

[31] Demirel A.H., Akgun A., Ongören A.U., Kisakurek M., Erol M.F. (2007). Unusally located hydatid cysts. J Acad Gastroenterol.

[32] Dogmus M., Kaplan M., Salman B., Yılmaz U. (2007). Unusual presence of hydatid disease in subcutaneous tissue: a case report. New J Med.

[33] Safioleas M., Nikiteas N., Stamatakos M., Safioleas C., Manti C.H., Revenas C. (2008). Echinococcal cyst of the subcutaneous tissue: a rare case report. Parasitol. Int..

[34] Parsak C.K., Eray I.C., Sakman G., Eray S.I., Gumurdulu D., Akcam T. (2008). Hydatid disease involvement of primary subcutaneous tissue in the posterior proximal thigh. An unusual localization. Int. J. Dermatol..

[35] Dirican A., Unal B., Kayaalp C., Kirimlioglu V. (2008). Subcutaneous cysts occurring in the palm and the tight: two case reports. J. Med. Case Rep..

[36] Gupta R., Mathur S.R., Agarwala S., Kaushal S., Srivastav A. (2008). Primary soft tissue hydatidosis: aspiration cytological diagnosis in two cases. Diagn. Cytopathol..

[37] Losanoff J.E., Richman B.W., Jones J.W. (2004). Primary hydatid cyst of the axilla. ANZ J. Surg..

[38] Savulescu F., Iordache I.I., Hristea R., Dumitru C., Sandru A.M., Balasa G. (2010). Primary hydatid cyst with an unusual location--a case report. Chirurgia (Bucur).

[39] Ozkan H.S., Sahin B. (2010). Primary hydatid disease of subcutaneous tissue in the leg. Clin. Exp. Dermatol..

[40] Singal R., Dalal U., Dalal A.K., Singh P., Gupta R. (2010). Subcutaneous hydatid cyst of the thigh. South. Med. J..

[41] Battyany I., Andrea L., Nagy K.K. (2011). Subcutaneous hydatid cyst in the popliteal fossa at the site of a previous wasp sting. Diagn. Interv. Radiol..

[42] Sallami S., Ayari K., Oueslati B., Miladi M. (2011). Isolated subcutaneous hydatid cyst. Tunis. Med..

[43] Ousadden A., Elbouhaddouti H., Ibnmajdoub K.H., Mazaz K., Aittaleb K. (2011). A solitary primary subcutaneous hydatid cyst in the abdominal wall of a 70-year-old woman: a case report. J. Med. Case Rep..

[44] Bansal C., Lal N., Jain R.C., Srivastava A.N., Fatima U. (2011). Primary hydatid cyst in the soft tissue of the face: an exceptional occurrence. Indian J. Dermatol..

[45] Mushtaque M., Mir M.F., Malik A.A., Arif S.A., Khanday S.A., Dar R.A. (2012). Atypical localizations of hydatid disease: experience from a single institute. Niger J Surg.

[46] Rais H., Jghaimi F., Bassi L., Ziad T., El Ganouni Cherif Idrissi, N, Belaabidia B. (2012). Hydatid cyst of the scalp. Rev. Stomatol. Chir. Maxillofac..

[47] Abhishek V., Patil V.S., Mohan U., Shivswamy B.S. (2012). Abdominal wall hydatid cyst: case report and review of the literature. Case Rep Surg.

[48] Jarboui S., Hlel A., Daghfous A., Bakkey M.A., Sboui I. (2012). Unusual location of primary hydatid cyst: soft tissue mass in the supraclavicular region of the neck. Case Rep. Med..

[49] Ozdemir G., Zehir S., Ozdemir B.A., Sipahioglu S., Severge U. (2012). Hydatid cyst involvement of shoulder and deltoid muscle: a case report. Eklem Hastalik. Cerrahisi.

[50] Vecchio R., Marchese S., Ferla F., Spataro L., Intagliata E. (2013). Solitary subcutaneos hydatid cyst: review of the literature and report of a new case in the deltoid region.

[51] Burgazli K.M., Ozdemir C.S., Beken Ozdemir E., Mericliler M., Polat Z.P. (2013). Unusual localization of a primary hydatid cyst: a subcutaneous mass in the paraumblical region. Eur. Rev. Med. Pharmacol. Sci..

[52] Almadani N., Almutairi B., Alassiri A.H. (2013). Primary subcutaneous hydatid wih palisading granulomatous reaction. Case Rep Pathol.

[53] Yucesoy C., Ozturk E., Hekimoglu B. (2013). Radiologic findings and percutaneous treatment of a rare giant soft tissue hydatid cyst. JBR-BTR.

[54] Haslak A., Uysal E. (2014). Left lumbar subcutaneous hydatid cyst disease: a case report. Turkiye Parazitol. Derg..

[55] Ekşi M.Ş., Bayri Y., Saraçoğlu A., Uyar Bozkurt S., Konya D. (Dec 2014). Primary subcutaneous hydatid cyst over thoracic spine: a case report and review of the literature. Turkiye Parazitol. Derg..

[56] Kayaalp Cuneyt, Dirican Abuzer, Aydin Cemalettin (2011). Int. J. Surg..

[57] Agha R.A., Fowler A.J., Saetta A., Barai I., Rajmohan S., Orgill D.P. (2016). for the SCARE Group, The SCARE Statement: consensus-based surgical case report guidelines. Int. J. Surg..

[58] M.P. Zarzosa, A. Orduna~ Domingo, P. Gutiérrez, P. Alonso, M. Cuervo, A. Prado, Evaluation of six serological tests in diagnosis and postoperative control of pulmonary hydatid disease patients, Diagn. Microbiol. Infect. Dis. 35 (December (4)) (1999) 255–262. 10.1016/s0732-8893(99)00079-6 10668582

[59] Zhang W., McManus D.P. (2006). Recent advances in the immunology and diagnosis of echinococcosis, FEMS Immunol. Med. Microbiol..

